# ResNet14Attention network for identifying the titration end-point of potassium dichromate

**DOI:** 10.1016/j.heliyon.2023.e18992

**Published:** 2023-08-06

**Authors:** Siwen Liang, Linfei Yin, Dashui Zhang, Dongwei Su, Hui-Ying Qu

**Affiliations:** a Guangxi Key Laboratory of Power System Optimization and Energy Technology, Guangxi University, Nanning, Guangxi, 530004, China; b School of Chemistry and Chemical Engineering, Nanning University, Nanning, Guangxi, 530004, China

**Keywords:** ResNet, Attention, Titration end-point, Potassium dichromate method, Deep learning

## Abstract

With the rapid development of industry, the increasing discharge of sewage causes the detection of water quality to be of increasing importance. Potassium dichromate titration is one of the most important testing methods in water quality detection; the ability to accurately identify the titration end-point of potassium dichromate is currently a research challenge. To identify titration end-point quickly and accurately, this study proposes a ResNet14Attention network, which utilizes residual modules that focus on original image information and an attention mechanism that focuses highly on classification targets. The proposed ResNet14Attention network is compared with 12 convolutional neural networks such as ResNet series networks, VGG, and GoogLeNet. The results of comparison experiments reveal that only the proposed ResNet14Attention network has the highest training and testing accuracy of 100% among all convolutional neural networks in the comparison experiment; the proposed ResNet14Attention network has the highest training speed compared to all the networks that over 90% accuracy.

## 1 Introduction

Recently, the rapid development of industry has contributed to massive sewage generation [1]. Substandard sewage treatment and discharge destroy water resources [2] and seriously affect the quality of human life [3] and ecological natural environments [4]. Both reduced ecological water quality pollution [5] and strengthen regulation of water quality are important measures to protect water resources [6]. Thus, water quality detection is an essential method of sewage regulation [7]. National standard law stipulates that chemical oxygen demand (COD) is applied to describe water pollution degree [8]. The COD, which is an important indicator for water quality detection results, reflects the water content of reductants and organics [9]. Currently, the potassium dichromate method, which is a common method to measure COD, applies the strong oxidizing ability of potassium dichromate to detect the organic content of sewage indirectly by chemical titration [10]. During the titration process of the potassium dichromate method, the ability to accurately identify the titration end-point strongly affects the accuracy of organic measurement results and the ultimate results of water quality detection. Since ions complete chemical reactions in a weak time, the changing critical range of the titration end-point is overly narrow, causing the titration end-point to be prone to be misidentified. For example, in the process of indicator-based titration, color may change quickly or imperceptibly, causing the identification of the titration end-point inaccurate [11]. Because the titration end-point is hardly accurately identified, the titration experiment is prone to over-titrate, resulting ultimate consumed solution volume being excessive, and eventually, the COD of sewage in the experimental is higher than the COD of sewage in practice, thus affecting the ultimate results of water quality detection. To ensure the authenticity and reliability of the experimental results, experimental operators generally elect to re-titrate. However, the re-titration inevitably consumes the time and effort of the experimental operator and wastes various assay reagents [12].

The existing methods for automatic identification of titration end-point can be divided into three main categories (Table 1), i.e., potential identification [13], color identification, and artificial intelligence identification. The potential identification method applies indicator electrodes to detect potential changes in solution reactions [14] and indicates titration end-point with potential abrupt changes [15]. The potential identification method is unaffected by solution color and turbidity, and merely relies on the potential abrupt changes to identify the titration end-point when ions react completely; the accuracy of potential identification method measurement is high [16]. However, the indicator electrode materials are expensive, hard to preserve, and require to be replaced for different chemical titration reactions. The accuracy of the potential identification method is low in the case of low ion concentration [17].

**Table 1:** Comparison of methods for identification of titration end-point.

Method	Principal	Advantage	Disadvantage
Potential identification [[13], [14], [15], [16], [17]]	Potential abrupt values in ionic reactions.	Unaffected by solution color and concentration; high detection accuracy.	Expensive electrodes; inconvenient to preserve; electrodes depend on different chemical reactions; high errors in low solution concentrations.
Spectral analysis [[18], [19], [20], [21]]	Wavelength absorbance depends on different colors.	High detection accuracy; fast detection speed; without touching solutions; safe operation.	Complex and expensive instrumentation; complex operation; require human determination of optimum measurement wavelength.
Color sensor identification [[22], [23], [24]]	Measure color based on three primary colors.	Convenient operation; instrument simplicity; fast detection speed.	Low accuracy; affected by the light environment; requires stable solution color changes.
Artificial intelligence identification [[25], [26], [27], [28], [29], [30], [31]]	Autonomous learning of convolutional neural networks.	Unaffected by environments; high detection accuracy; fast detection speed; excellent sensitivity; convenient operation.	Long training time.

Color identification methods identify titration end-point based on the color change of indicators or reaction solutions. According to the method of judging color abrupt change, color identification can be divided into two categories. One category applies spectral analysis, which is a method based on the Lambert-Bier principle [18], to identify the titration end-point by the different absorbance of wavelength caused by color change [19]. The spectral analysis method judges color change from the perspective of optics with high accuracy and avoids contact with harmful chemicals such as sulfur dioxide for ensuring the safety of operators [20]. However, the spectral analysis method is complicated to operate, and requires huge instrumentation and equipment; the optimal absorption wavelength requires to be set for different solution reactions [21]. The second category of color identification method is color sensor identification, which judges the color abrupt change based on three primary colors (i.e., red, green, and blue) that are collected by photoelectric elements, thus determining the titration end-point [22]. The color sensor is a simple instrument with rapid color identification speed; the environmental applicability of the color sensor is strong, allowing the settings of the sensor to be adjusted with environmental changes [23]. However, the color sensor requires stable ambient light and stable color change of solution during operations. Otherwise, the accuracy of the color sensor for titration endpoint identification may be decreased by the environmental factors of the light and the solutions [24].

Artificial intelligence identification, which replaces the human eye with machine vision [25], is the third method to automatically identify titration end-point. Recently, convolutional neural networks (CNNs) have performed excellently in computer vision [26] and are widely applied in various fields [27]. The CNNs extract image features to self-learn through different structures of hidden layers and obtain quite an effective classification identification [28]. Especially for some classification identification of subtle changes in images, such as medical images [29,30] and the analysis of plant symptoms [31], the CNNs have outstanding advantages. In medical image classification, deep ensembles that benefit from a wide range of architectural advances are continuously proposed to improve classification performance [32]. In plant monitoring, an increasing number of machine learning techniques are applied to crops’ growth and leaf diseases [33]. In particular, deep network ensembles excel in performing hyperspectral data classification and unmixing in the case of high volume of hyperspectral image data [34]. For example, ResNet series networks adopt residual module structure with skip connection, enabling networks to learn more original information in the forward transmission and alleviating degradation problems caused by excessive information loss [35]. As the layers of the CNNs are stacked, the training of networks may appear gradient explosion and the gradient vanishing. The ResNet series networks skillfully apply batch normalization (BN) between convolutional layers to satisfy the same distribution pattern of feature maps, thus avoiding the gradient explosion and the gradient vanishing, and improving the accuracy of classifying identifications [36]. In addition, the attention mechanism has been extensively applied in the fields of image processing [37], natural language processing [38], and data prediction [39]. Traditional CNNs incorporating attention mechanisms can enhance network performances. For example, during classification detection in CNNs, the attention mechanism can enhance attention to focus on classification target objects, weaken irrelevant information, and extract valuable detailed information [40]. Because the color change of potassium dichromate titration is subtle and quick, machine vision classification identification presents the challenge that the similarity of solution images between different categories may be high, while the difference of solution images within the same category may be strong. Inspired by the high ability of ResNet series nets to process original information and the high focus of attention mechanism on targets, this study proposes a ResNet14Attention network, which optimizes ResNet18 network into ResNet14 network and incorporates attention mechanisms for identifying the titration end-point of potassium dichromate titration detection. The research contributions of this study are presented in the following four primary aspects.

(1) To the best knowledge of the authors, this study presents a first modified simple deep CNN method to solve inaccurate identification of titration end-point in chemical titration reactions. This study optimizes the ResNet18 network and incorporates attention mechanisms for the titration end-point identification of potassium dichromate. Compared with other titration end-point identification methods, CNN with image classification can minimize the identification errors of instruments and human operations, reduce the expense of complex instruments, and improve the accuracy of titration end-point identification.
(2) When identifying titration end-point by color sensors or electrodes, solution concentration and ambient light will affect ultimate titration detection results. In contrast, this study employs a CNN to acquire solution images containing environmental factors, fully utilizes the whole image information for convolutional processing to obtain comprehensive and reliable titration detection results, and reduces the effect of environmental factors on titration end-point identification.
(3) Compared with traditional CNNs, this study proposes a simpler deep neural network that combines the advantages of the ResNet18 network and attention mechanisms, thus greatly enhancing network performances. The backbone structure of the proposed ResNet14Attention network is optimized from ResNet18 to ResNet14 by reducing four convolutional layers, thus reducing system running memory and speeding up the training and testing of the model, and obtaining a relatively high accuracy rate in experiments.
(4) The proposed ResNet14Attention network incorporates attention mechanisms in ResNet14, contributing to focus the attention of the network on image subtle changes, avoiding the loss of critical information, and combining global information and local details to train in the learning process, thereby can improve the identification accuracy of images for accurately identifying the titration end-point of potassium dichromate.

The rest structure of this study is arranged as follows. The description of the potassium dichromate titration process is in Section 2. The structure and framework of the ResNet14Attention are presented with details in Section 3. The presentation and analysis of comparison experimental results between ResNet14Attention and other CNNs are discussed in Section 4. The summary of this study is described in Section 5.

## 2 Potassium dichromate titration sample collection and pretreatment

**Fig. 1:**
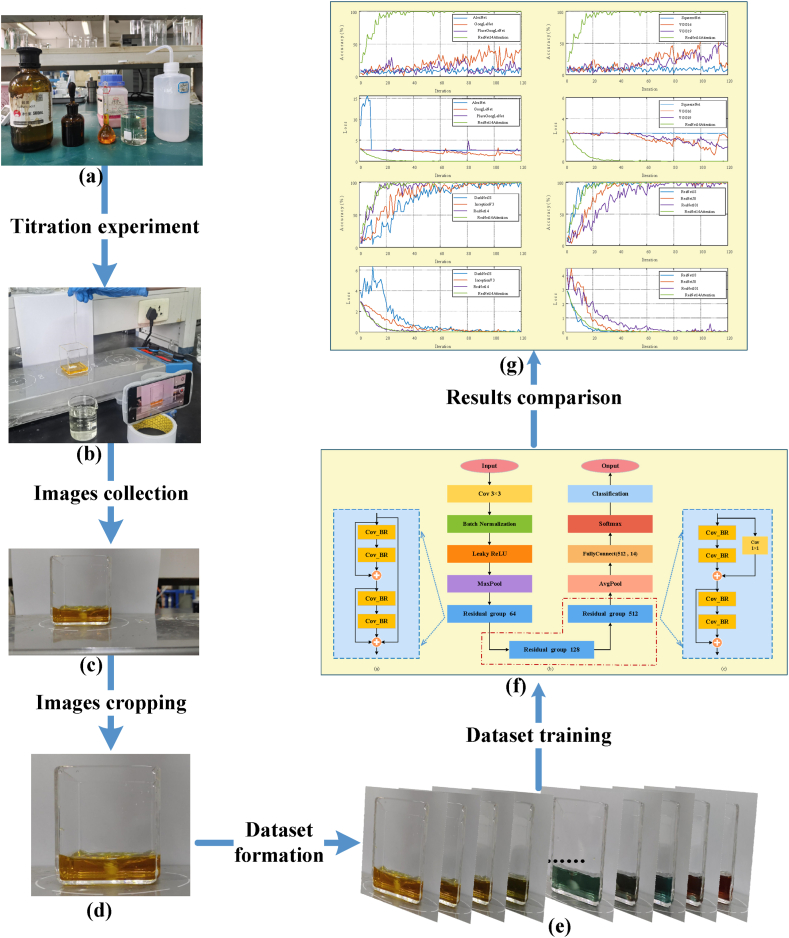
Experiment and simulation steps: (a) reagent preparation; (b) sample sampling; (c) unprocessed samples; (d) cropped samples (e) formation of the dataset; (f) training of the proposed network; (g) obtaining training and testing results.

The steps of experiments and simulations in this study are shown in Fig. 1(a–g). Images collected from the potassium dichromate titration experiment are applied as the dataset of CNNs to train and test.

The majority identification of chemical titration end-point are identified by the naked vision of operators for simple and economical chemical experiments, resulting in relatively high subjective errors. In this study, machine vision is applied to replace naked vision, reducing subjective errors. To acquire data samples for machine vision learning, this study conducts a potassium dichromate chemical titration experiment and captures images of solutions at the titration process by a camera. Sample acquisition of potassium dichromate titration consists of two parts, chemical experiment and image acquisition/processing.

In the chemical experiment of potassium dichromate titration, experimental reagents consist of 0.125 mol/L standard solutions of potassium dichromate, 0.1 mol/L solution of ferrous ammonium sulfate, ferroin indicator solution, concentrated sulfuric acid, and deionized water. The chemical reagents utilized in the chemical experiment were purchased from qualified manufacturers in the market. The titration process is listed as follows: take 2 mL of potassium dichromate solution in a beaker; add about 20 mL of deionized water for diluting the potassium dichromate solution; put a magnetic stirring bar in the beaker and turn on a magnetic stirrer for stirring; add 1.25 mL of concentrated sulfuric acid to the reaction beaker with a pipette gun for keeping the potassium dichromate solution strongly acidic; then, after the solution cooling, add two drops of ferroin indicator solution to the potassium dichromate solution for reaction indicator. The titration process shown in Fig. 1 (b) is titrated by ferrous sulfate solution with a test tube. During the titration process, the color of the solution changes from yellow through blue-green to reddish-brown. The titration end point means that the solution shows a stable reddish-brown color.

The chemical titration process of potassium dichromate is recorded by a fixed camera with 1920 × 1080 pixels and a 29.98 fps frame rate of videos. Then, all the images recorded in the video are extracted as a sample according to the frame interval of three. All the samples are divided into 14 categories and stored in folders, noted as original images (Fig. 1(c)). To improve classification accuracy, the original images are cropped with 931 × 840 pixels, and recorded as cropped images (Fig. 1(d)).

**Fig. 2:**
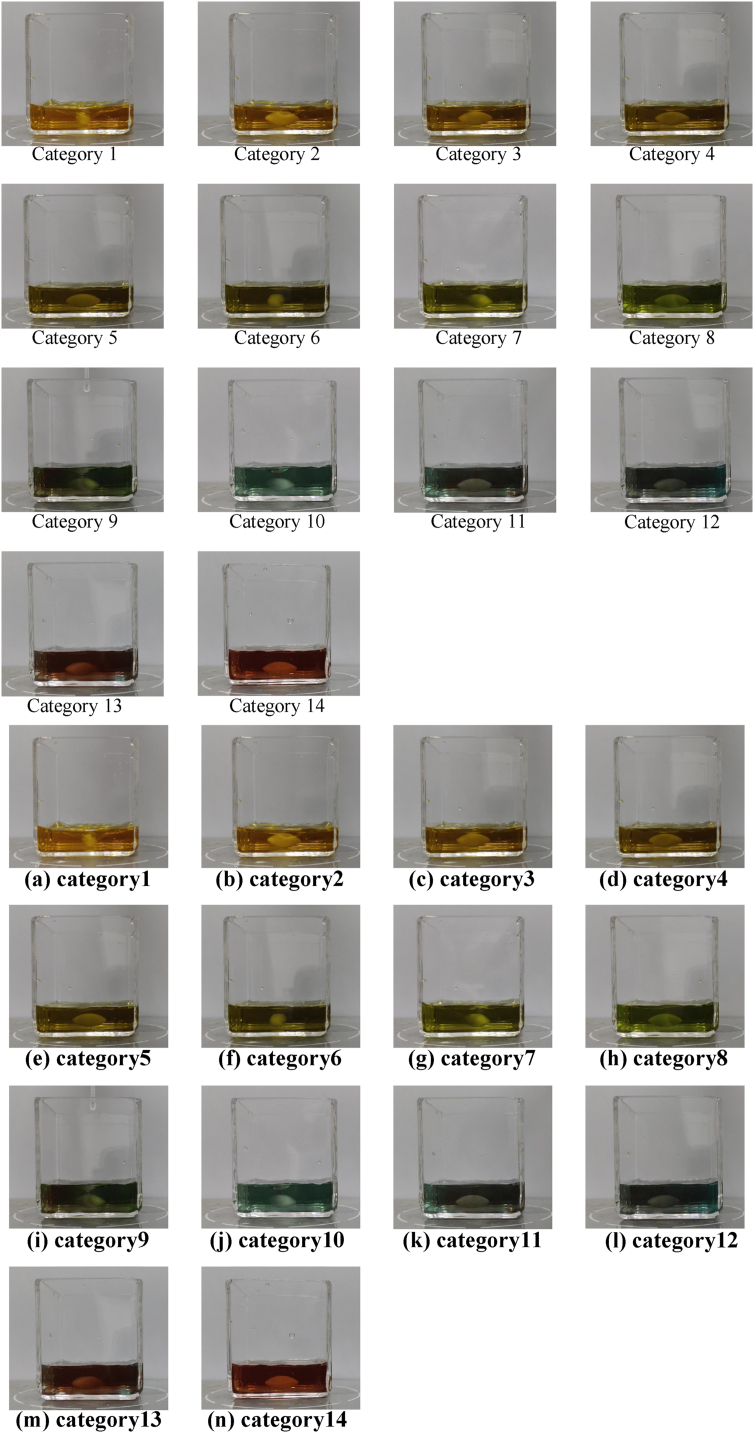
Images of 14 categories of reaction solutions: (a) category 1; (b) category 2; (c) category 3; (d) category 4; (e) category 5; (f) category 6; (g) category 7; (h) category 8; (i) category 9; (j) category 10; (k) category 11; (l) category; (m) category 12; (n) category 14.

**Fig. 3:**
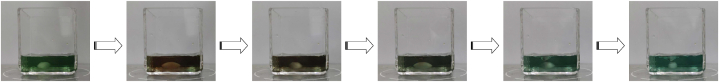
Images in category 8.

During the titration process, solution color changes constantly; how to quickly and accurately identify the titration end-point is the focus of this study. Among the 14 categories of solution images (Fig. 2(a–n)), the change of color in non-adjacent categories is relatively clear. For example, the images of Category 1 (Fig. 2 (a)) and Category 10 (Fig. 2 (j)) can be identified with naked vision. However, the change of color in adjacent images is relatively subtle, especially when the titration end-point is approximately reached. For example, from Category 11 to Category 14 (Fig. 2(k–n)), the solution appears in the same color as the titration end-point; Categories 11 and 12 (Fig. 2 (k, l)) are presented locally; Category 13 (Fig. 2 (m)) really reaches the titration end-point; while Category 14 (Fig. 2 (n)) is already over-titrated. Since chemical reactions are continuous and rapid, the images of the same category may even differ substantially. For instance, Category 8 (Fig. 2 (h)) shown in Fig. 3 has different images with time variations in the titration process.

In the process of human titration, if naked vision is slightly inattentive and experimenters cannot distinguish which stage the solution reaction proceeds, then, titration is prone to over-titration and the consumed volume of the titration solution is inaccurate, thus failing the titration experiment. To ensure the accuracy of detection, experimenters are required to repeat the titration experiment, thus wasting both reagent solutions and the time and energy of the experimenters. Therefore, this study proposes a novel machine vision method for the accurate identification and prediction of titration end-point.

## 3 The proposed ResNet14Attention

The proposed ResNet14Attention network applies residual modules to focus on original image information and incorporates attention mechanisms to focus on classification targets.

**Fig. 4:**
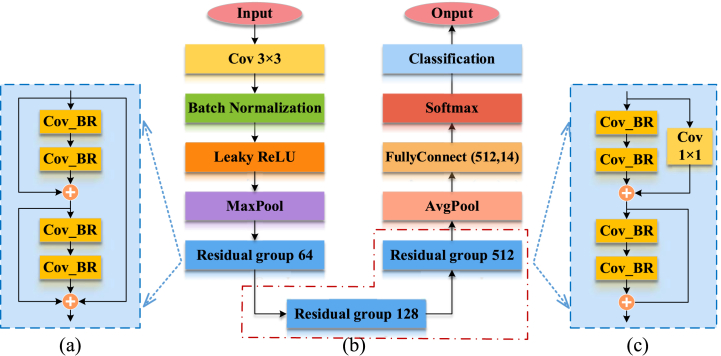
Module of the proposed ResNet14Attention: (a) attention skip residual; (b) ResNet14Attention; (c) residual module groups with 128 and 526 channels.

**Fig. 5:**
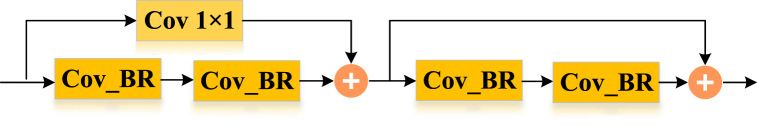
Residual module group.

The proposed ResNet14Attention network (Fig. 4 (b)) is optimized from the ResNet18 to ResNet14 networks. Compared with the backbone structure of the ResNet18 network, the difference of the backbone structure of the ResNet14Attention network is the reduction of one residual module group (Fig. 5) with 256 channels, i.e., the reduction of four convolutional layers. The proposed ResNet14Attention network applies only the residual module group of three different channels, i.e., 64 (Fig. 4 (a)), 128, and 526 channels (Fig. 4 (c)). Compared with the ResNet18 network, the ResNet14Attention network contains fewer layers, meaning less system running memory, thus increasing training speed. The ConV_BR of Fig. 5 represents the 3 × 3 convolutional layer, BN, and rectified linear unit (ReLU) activation function.

**Fig. 6:**
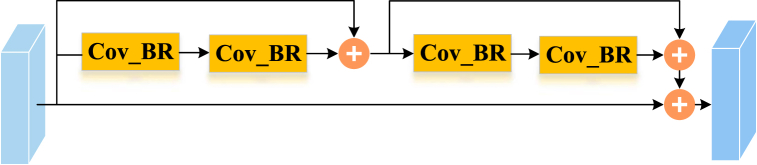
Attention skip residual.

Inspired by the attention mechanism for calculating weights and residual module learning, the ResNet14Attention network introduces the attention skip residual module (Fig. 6) after the first convolutional layer and maximum pooling layer. The parallel association of two tandem residual module groups and one identity mapping forms the attention skip residual module in the ResNet14Attention network. The attention skip residual module calculates weights and attention values from the two residual module groups; then, the output of the two residual module groups is superimposed with the output of the identity mapping, i.e., the two output matrices are summed by elements. The superimposed matrix is regarded as the input of the next residual module group. The input pays higher attention to the original image and enhances the attention to details in the next feature learning process.

### 3.1 ResNet of ResNet14Attention

**Fig. 7:**
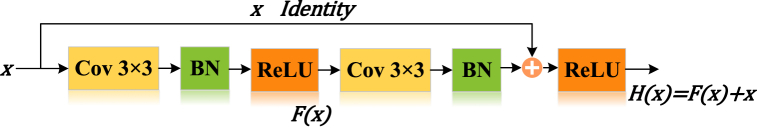
Residual model.

The core of ResNet in the proposed ResNet14Attention is the residual module (Fig. 7), which is designed primarily to solve degradation problems and gradient exploding or gradient vanishing in the deepening of networks, thus focusing on more original image information [41].

Since each network has an unknown optimal number of layers to train for different samples, redundant layers may occur when training a network with a fixed number of layers. Therefore, as the depth of the networks increases, the redundant layers will learn extra parameters with the loss of original information, causing model degradation. When redundant layers are identified, applying constant mapping, i.e., ensuring input values and preset values are constant, can avoid the impact of extra parameters in redundant layers. However, the output value equal to the input value is quite hard by learning network layers. To solve the problem of that redundant layer input and output that are hard to equal, the residual module (Fig. 7) applies the method of residual value superposition. The residual value is a difference value between the preset value and the input value: *F*(*x*) = *H*(*x*)-*x*, where x is the input value, and *H*(*x*) is the preset value. Hence, the final mapping output value of the residual module is *F*(*x*) = *H*(*x*)+*x*. The residual module connects the input value *x* directly to the output with a skip connection, i.e., a simple shortcut, while the residual value *F*(*x*) is learned by layers. When identifying a redundant layer, the residual module learns a zero residual value: *F*(*x*) = 0, meaning that the output of the redundant layer is constantly equal to the input, corresponding to a redundant layer without network effect, thus, solving the degradation problem of deep networks [42]. Compared with directly learning the output and input values to be constant: *H*(*x*) = *x*, learning *F*(*x*) = 0 is relatively simple, because the initial parameter values of the network in each layer are generally inclined to zero. Consequently, the redundant layer learning the parameter with *F*(*x*) = 0 can achieve rapid convergence.

In addition, when the residual module is identified as a non-redundant layer, skip connection can send original input information *x* forward and add to the after-learning information without processing, allowing the detailed information of the shallow layer to feed directly into deep layer networks, thus the deep layer networks learning can focus on original image information.

**Fig. 8:**
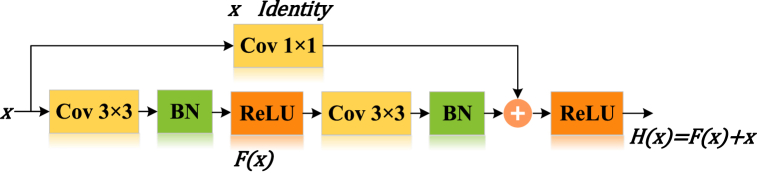
Residual model with ascending convolution layer.

To address the parameter mismatch between different channels, a 1 × 1 convolutional layer (Fig. 8) is applied to the channel of skip connection to increase dimensionality without reducing feature information [43]. Therefore, the feature values of different channels can be summed up directly.

**Fig. 9:**
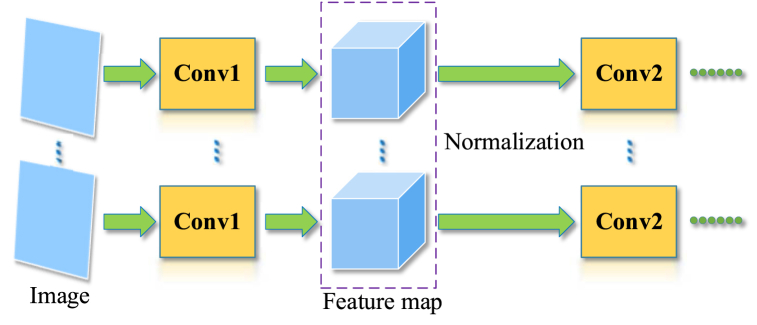
General normalization of networks.

Additionally, the residual module utilizes BN to speed up the network training. In image preprocessing, the images are generally normalized for accelerating the convergence of networks [44]. In general network normalization (Fig. 9), after the input of the image passes through two convolutional layers, the feature map of each image may not satisfy the same distribution law. If normalization is performed directly on the feature map of whole training sets, the calculation of networks will be particularly huge, thus ensuring the convergence of the network while increasing training time [45].

The BN is a batch processing of images in different channels. The feature map is normalized in a batch of the same channel, leaving the distribution of channel data with a mean value of zero and a variance of one [46]. For example, the input of a red-green-blue color image is normalized in three channels, i.e., the red channel, green channel, and blue channel. In the same channel, all feature map elements are served as the BN input: $X=\left\{x^{\left(1\right)},x^{\left(2\right)},\ldots \ldots ,x^{\left(n\right)}\right\}$; where $x^{\left(n\right)}$ denotes a feature vector of *n*th image. Input image size is w×h; the element number of X vector is m=w×h×n. Firstly, seek mean value: $\mu_{X}\leftarrow \frac{1}{m}\sum_{i=1}^{m}x_{i}$; secondly, seek variance: $\sigma_{X}^{2}\leftarrow \frac{1}{m}\sum_{i=1}^{m}{\left(x_{i}-\mu_{X}\right)}^{2}$; eventually, obtain regularized output: ${\hat{x}}_{i}\leftarrow \frac{x_{i}-\mu_{X}}{\sqrt{\sigma_{X}^{2}+\varepsilon }}$. Then, the output ${\hat{x}}_{i}$ presents a distribution with a mean value of zero and a variance of one. Therefore, all the feature maps obtained by BN satisfy the same distribution law, thus improving training speed and stabilizing training effects as the layers of neural networks increase.

The transmission process with BN is: $f_{l}=BN\left(f_{l-1}W_{l}\right)=\frac{1}{\sigma_{l}}\left(f_{l-1}W_{l}-\mu_{l}\right)$. Backpropagation is: $\frac{\partial f_{l}}{\partial f_{l-1}}=W_{l}\frac{1}{\sigma_{l}}$. Thus, gradient backpropagation of successive multilayers is $\frac{\partial f_{l}}{\partial f_{k}}=\prod_{i=k+1}^{l}W_{l}\frac{1}{\sigma_{l}}$. The standard deviation matrix $\frac{1}{\sigma_{l}}$ scaled by BN for the weights $W_{l}$ keeps the output of each layer satisfying the same distribution law, thus avoiding the problem of gradient vanishing and exploding in network training [47].

**Fig. 10:**
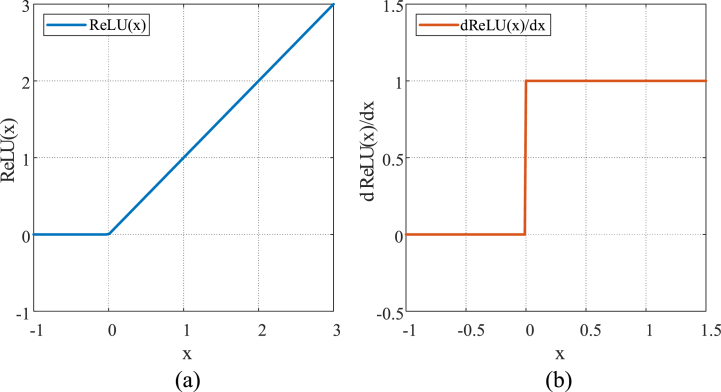
ReLU and its differential: (a) ReLU; (b) the differential of ReLU.

Moreover, the residual module utilizes the ReLU activation function (Fig. 10(a)): $Relu\left(x\right)=\max\left(x,0\right)=\left\{ \begin{array}{c} 0,x<0\\ x,x>0 \end{array}\right.$. When x>0, the differential of Relu(x) (Fig. 10(b)) is constantly equal to one, meaning that the ReLU activation function can avoid the problem of gradient exploding and gradient vanishing when continuous multiplication occurs in deep networks. When x<0, the differential of Relu(x) is constant equal to zero, meaning that the outputs of some neurons are zero. Thus, the network is sparse and the interdependence of parameters is reduced, slowing down the problem of overfitting to a certain extent, increasing the computational speed, and speeding up network training. Compared with Sigmoid and Tanh functions, the ReLU activation function tunes neuron activity by linear correction and regularization, leading to more efficient backpropagation, more decentralized activity, a simpler computational process, and lower time cost [48].

**Fig. 11:**
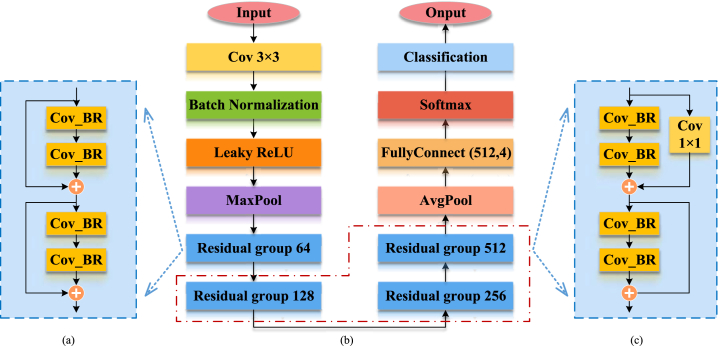
ResNet18 network: (a) Residual module group with 64 channels; (b) ResNet18 network; (c) Residual module groups with 128, 256, and 526 channels.

The ResNet18 (Fig. 11(b)) network consists of eight residual modules directly connected to form four residual module groups. Since the residual modules of the ResNet18 network consist of four different channels (i.e., 64, 128, 256, and 512 channels), a 1 × 1 convolutional layer (Fig. 11(c)) is applied in the residual module group for the other three channels except the 64 channels (Fig. 11(a)) to achieve the function of upscaling and superposition.

### 3.2 Attention mechanism of ResNet14Attention

The core target of attention mechanisms is to focus on critical information and suppress useless information. Specifically, the attention mechanism simulates human vision, i.e., visual focus, for filtering out critical information, selecting the most important information from total information, focusing feature learning attention on focus targets to obtain more useful and detailed information, and reducing the influence of useless information or ignoring irrelevant information. The attention mechanism expresses the degree of information attention by weights, with high weights indicating focus on important information and low weights indicating neglect of irrelevant information. Therefore, the efficiency and accuracy of visual information processing are highly improved by the guidance of attention mechanism weights. In different situations, the attention mechanism can determine different important information by adjusting the weights to satisfy the requirements of different focus objects [49].

**Fig. 12:**
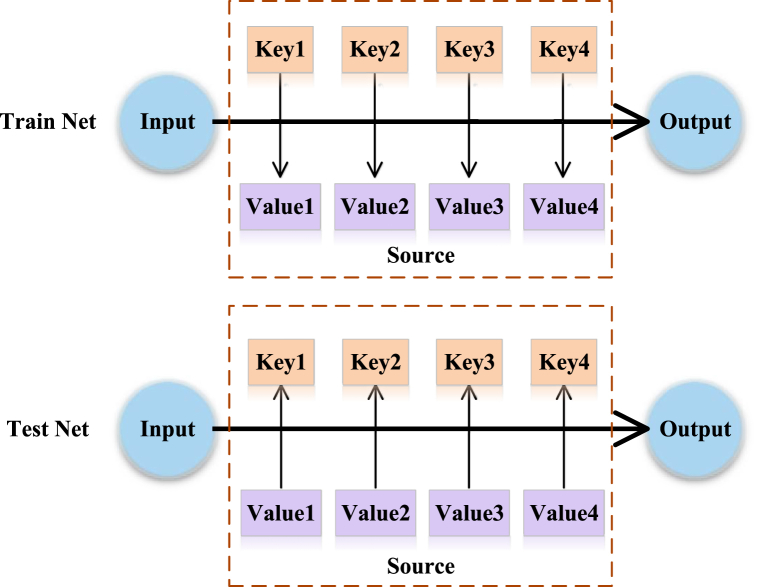
Basic network model of attention mechanism.

The basic network framework in Fig. 12 shows the principle of attention mechanisms. The essence of the attention mechanism is to calculate Value weighting coefficients based on Input and Key and sum the Value of elements in a Source. The Source consists of a series of Key and Value data pairs. During data training, firstly, the Input is given, then, the correlation between the Input and each Key is calculated, the weight coefficient of each Key corresponding to the Value is obtained, and eventually, the Value is summed up to obtain the final attention value. During data testing, the Input and each Key can adjust the corresponding learning attention size according to the weight coefficients obtained in training [50].

## 4 Case studies

This study of potassium dichromate titration experiment in Classroom 301, Building 2, School of Chemistry and Chemical Engineering. Besides, the proposed algorithm is completed in the processor of Intel 8160 CPU, 128 GB memory with Windows 10 system. The proposed network training software for this work is MATLAB R2021b.

**Fig. 13:**
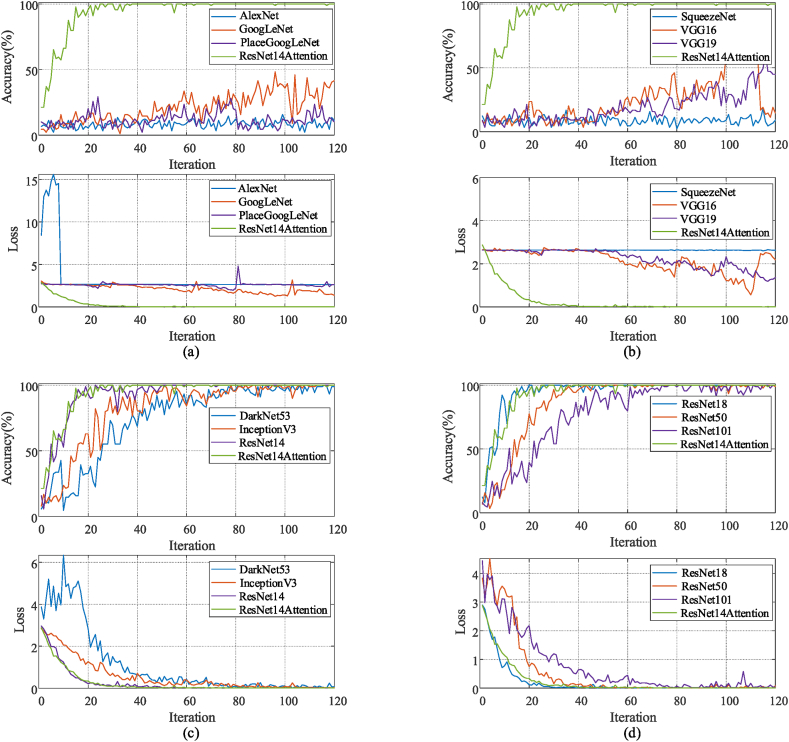
Accuracy and loss curves of 13 CNNs: (a) AlexNet, GoogLeNet, Place365GoogLeNet, the proposed ResNet14Attention; (b) SqueezeNet, VGG16, VGG19, ResNet14Attention; (c) DarkNet53, InceptionV3, ResNet14, ResNet14Attention; (d) ResNet18, ResNet50, ResNet101, the proposed ResNet14Attention.

**Fig. 14:**
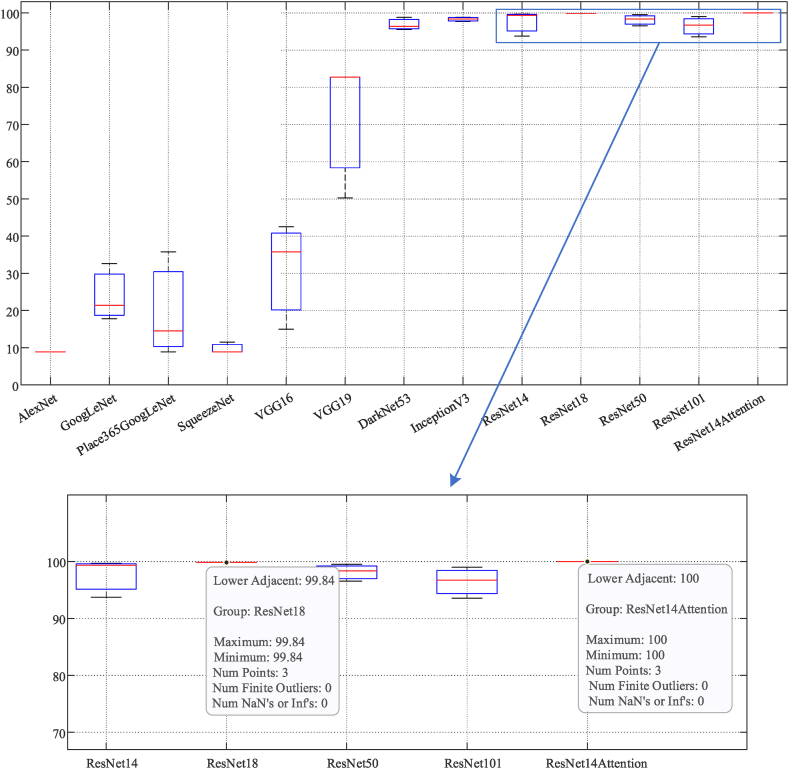
Boxplot of test accuracy of ResNet14Attention and other networks.

The experiments in this study train and test a total of 13 CNNs, including AlexNet, GoogLeNet, Place365GoogLeNet, SqueezeNet, VGG16, VGG19, DarkNet53, InceptionV3, ResNet14, ResNet18, ResNet50, ResNet101, and the proposed ResNet14Attention. The total number of image sample datasets is 2033; 70% of the dataset is for training the compared CNNs; 30% of the dataset is for validating trained CNNs. Since this study divides the potassium dichromate titration process into 14 categories, the neuron number of output depth fully connected layer of each network is set to 14. In the training settings, the solver is stochastic gradient descent with momentum, the initial learning rate is 0.01, the validation frequency is 89, the maximum epoch (MaxEpoch) of training is 8, and the minimum batch size (MiniBatchSize) of each training epoch is 16. The ultimate curves of training accuracy and loss function are obtained from all networks trained according to the above settings (Fig. 13(a–d)). The ultimate comparisons of accuracy and training time are obtained from all networks trained and tested (Table 2). After several testing sessions, the test accuracy of ResNet14Attention and other networks is cross-validated with a boxplot (Fig. 14).

**Table 2:** Statistical results of 13 networks. (Bold represents optimal results with 100% training accuracy).

Network	Image size	Layers	Connections	Train accuracy (%)	Test accuracy (%)	Training time (s)
AlexNet [51]	227	25	24	8.84	8.90	285
GoogLeNet [52]	299	170	181	27.07	32.62	1563
Place365GoogLeNet	224	144	170	13.81	14.50	1396
SqueezeNet [53]	227	69	76	8.84	8.90	963
VGG16 [54]	224	41	40	75.74	79.94	4237
VGG19 [55]	224	47	46	53.16	50.25	4958
DarkNet53 [56]	256	184	206	99.44	96.38	4319
InceptionV3 [57]	299	315	349	99.93	98.85	4079
ResNet14	224	55	60	**100**	93.74	1135
ResNet18 [58]	224	71	78	**100**	99.84	1213
ResNet50 [59]	224	177	192	**100**	96.54	4290
ResNet101 [60]	224	347	379	**100**	93.57	5683
Proposed ResNet14Attention	224	55	61	**100**	**100**	**1088**

In this experiment, the high accuracy of the ResNet14Attention network indicates that the ResNet14Attention network combines the advantages of the residual module and attention mechanism, exhibiting higher attention and focus on both original and detailed information, and achieving accurate classification identification with minimum time. Among all experimental networks, the ResNet14Attention network has the highest classification accuracy and the shortest training time among all the networks with test accuracy >50%.

Some shallower networks, such as AlexNet with 25 layers, VGG16 with 41 layers, and VGG19 with 47 layers, have the insufficient depth for feature learning and are learned inadequately. While some deeper networks, such as GoogLeNet with 170 layers and Place365GoogLeNet with 144 layers, learn excessive depth. Hence, the deeper networks lost much information in feature learning during learning and do not capture the detailed information of titrated images. Therefore, neither excessive layers nor insufficient layers are trained with high accuracy, not conducive to the classification and identification of potassium dichromate titration images. The networks with deeper than 170 layers such as DarkNet53 and InceptionV3 can achieve more than 90% training accuracy because the number of connections in the networks is sufficient. However, the DarkNet53 network and InceptionV3 network obtain high accuracy at the expense of huge training time. Compared with other networks, the proposed ResNet14Attention network utilizes a suitable number of residual modules and a simple attention mechanism to ensure the learning extent of titrated image information while avoiding the loss of original information, greatly improving the ability to focus on image details. Moreover, the simple ResNet14Attention network reduces computational cost and training time, improves training speed, and facilitates rapid identification of potassium dichromate titrations.

Since the proposed ResNet14Attention network is inspired by ResNet18, this study conducted a comparative experimental analysis for the ResNet series of networks. The experimental results of the ResNet series networks show that all the training accuracy can achieve 100%, however, the highest test accuracy can only achieve 99.84%. In particular, the ResNet14 network only contains less attention mechanism than the ResNet14Attention network, yet the test accuracy of ResNet14 only achieves 93.74%. In addition, the training speeds of the ResNet series networks are lower than that of the ResNet14Attention network. Therefore, the proposed ResNet14Attention has higher training speed in training and higher test accuracy in testing compared to ResNet series networks.

Therefore, among the networks that achieved more than 90% accuracy in both training and testing, the ResNet14Attention network has the highest training speed and the least training time; the highest training and testing accuracies are both 100% obtained by the proposed ResNet14Attention network.

Cross-validation of ResNet14Attention network and other networks is performed in this study. The boxplot (Fig. 14) verifies that the proposed ResNet14Attention network has a higher and steadier test accuracy compared to the other networks. The test accuracy of ResNet series networks has higher value but not the highest value, with a wide variation range and less stable than that of the proposed ResNet14Attention network.

The test accuracy of multiple experiments is checked for p-value to obtain a p-value of 7.5906 × 10^−17^. The resulting p-value is far less than 0.05, indicating that the probability of a small event occurring is lower and results are more significant. Therefore, the test accuracy of this experiment is highly credible.

During the potassium dichromate titration, the solution color and volume changes are subtle because the ions react quickly and the amount of titrant per drop is small. Furthermore, when another ion is present in the chemical reaction, the solution changes instantaneously to another color in the presence of an indicator. The change between the two different colors is rapid, especially at the end of the critical titration. Additionally, the concentration of ions involved in the reaction is relatively low. Thus, the color changes quite quickly. Moreover, the titration is not sufficiently stirred within a short time. Then, the solution will partially reach the titration end-point resulting in an image with a different uniformity of color. Consequently, in the early stage of titration, the solution images of adjacent classes may not differ strongly from each other. However, in the later stages of titration, the solution images of different moments in the same category may differ remarkably. Therefore, the method of identifying titration end-point by artificial intelligence has the great advantage of being sensitive to the subtle performance of solution changes and being able to improve the attention and focus on the image details, thus increasing identification accuracies.

The parameter settings of MATLAB learner employed for training and testing in this study are described as follows. Primary parameters employed for the learner settings include validation frequency, MaxEpoch and MiniBatchSize. One MaxEpoch is a complete traversal of a training algorithm over whole training datasets. One MiniBatchSize is the number of images in each iteration. The training dataset in this study has 1428 images, which is a small number compared to existing publicly available datasets. Therefore, experimental results can attain stable values in a short number of training epochs (4–12). After several experimental trials, the MaxEpoch selected for this study is 8. Considering the small number of training datasets, the min-batch is normally selected to be small, ranging from 4 to 64, with a priority on the power of 2, to achieve sufficient utilization of the datasets and less wasted datasets. Therefore, the MiniBatchSize in this study is selected as 16. According to the number of training 1428 and the selected values of MaxEpoch and MiniBatchSize, the number of each iteration is calculated as 89.5. The iterative results present a trend as the number of iterations increases and ultimately reach a stable value. To visualize the trend and to consider verification frequency with an equal number of verifications in each iteration, the number of each iteration multiplied by a value no greater than 1 is rounded up to the validation frequency. For example, if the value 0.5 is multiplied by 89.5 and rounded up, the validation frequency is 44, i.e., a training epoch is validated twice. In this study, the validation frequency is 89, i.e., one training epoch is validated once. The parameter settings selected in this study not only ensure the correctness of the experiment, but also reasonably decrease the overall time for testing all networks.

The deficiencies of this study are summarized as follows: (1) The proposed ResNet14Attention is mainly oriented to the titration images of potassium dichromate; other chemical titration images have not been experimentally compared. (2) The proposed ResNet14Attention training time has space for improvement to accommodate faster changes in chemical titration reactions. (3) The proposed ResNet14Attention has a relatively limited number of classifications, i.e., only 14 categories; the proposed network can further study the accuracy for classifying more classified titration end-point.

## 5 Conclusions

For the problem that potassium dichromate titration is hard to accurately identify the titration end-point caused by the fast change of titration, the high similarity of images in different categories, and the large difference of images in the same category, the ResNet14Attention network is proposed. In this study, 13 networks are trained with 1428 images and tested with 607 images. The comparison results show that the proposed ResNet14Attention network has the highest accuracy rate, with 100% accuracy in both training and testing. In addition, the proposed ResNet14Attention network has the highest training speed among all networks with an accuracy of over 90%. The advantages of the proposed ResNet14Attention network are summarized as follows.

(1) This study proposes an optimized ResNet14Attention network incorporating residual modules as the main network structure and attention mechanisms. The ResNet14Attention network is applied for the classification identification of potassium dichromate titration end-point for the first time. The ResNet14Attention utilizes six residual modules to reduce information loss and focus more on original information and incorporates the attention mechanism to improve focus on main targets, greatly improving the ability to process details of potassium dichromate images. Compared with the other 12 CNNs, experiments show that the proposed ResNet14Attention has the highest classification accuracy and the highest classification speed with 100% accuracy in both training and testing and consumes the least training time among all networks with test accuracy over 90%.
(2) The proposed ResNet14Attention network applies the residual module to solve the problem of gradient exploding or gradient vanishing in the networks, alleviating the degradation problem of losing excessive information during deep learning, and allowing more original image information to be incorporated into the deep network. Moreover, by incorporating the attention mechanism, the proposed ResNet14Attention focuses the attention on targets in training and testing, is sensitive to the subtle solution changes in the potassium dichromate titration process, reduces the influence of environmental factors, and obtains a relatively high classification accuracy.
(3) Both BN processing and ReLU activation functions in the residual module of the proposed ResNet14Attention have the advantages of simplifying the computational process and reducing the computational cost, improving training speed to a certain extent. Moreover, the attention mechanism in the proposed ResNet14Attention provides a focus extent reference to deep networks in processing information by calculating the weight of attended targets, thus reducing the attention of useless information and greatly improving the speed of training and testing. Therefore, the proposed ResNet14Attention network is the most rapid in classification identification among all experimental networks and satisfies the requirement of fast identification of potassium dichromate titration end-point.

For future research, more accurate, less system memory, simpler, and with faster training networks could be proposed for potassium dichromate titration classification identification. Besides, more attention mechanisms could be incorporated for improving the training speed of networks. Furthermore, the proposed network could be applied to different chemical titration reactions to verify applicability. In addition, more efficient training functions with better convergence performances could be proposed for more classifications of titration images.

## Author contribution statement

Siwen Liang: Conceived and designed the experiments; Performed the experiments; Analyzed and interpreted the data; Wrote the paper.

Linfei Yin: Conceived and designed the experiments; Performed the experiments; Analyzed and interpreted the data; Contributed reagents, materials, analysis tools or data; Wrote the paper.

Dashui Zhang; Hui-Ying Qu: Contributed reagents, materials, analysis tools or data.

Dongwei Su: Performed the experiments.

## Data availability statement

Data will be made available on request.

## Declaration of competing interest

The authors declare that they have no known competing financial interests or personal relationships that could have appeared to influence the work reported in this paper.

## Appendix Nomenclature

**Variables**
$\sigma_{l}$: Inverse of the standard deviation matrix
$f_{l}$: Function of the *l*th batch normalization transmission process
*F*(*x*): Residual value of residual module
*h*: Height of batch normalization input image
*H*(*x*): Preset value of residual module
εA number tending to infinity*n*: A number tending to infinity*n*Number of batch normalization input image
*m*: Element number of X vector
*x*: Input value of residual module
${\hat{x}}_{i}$: Input of batch normalization
$x^{\left(n\right)}$: Feature vector of *n*th image
*X*: Input of batch normalization
*w*: Width of batch normalization input image
$W_{l}$: Weights of batch normalization
$\mu_{X}$: Mean value of batch normalizationAbbreviations
BN: Batch Normalization
COD: Chemical oxygen demand
CNNs: Convolutional neural networks
ReLU: Rectified linear unit
MaxEpoch: Maximum epoch
MiniBatchSize: Minimum batch size
